# Are institutional deliveries equitable in the southern states of India? A benefit incidence analysis

**DOI:** 10.1186/s12939-024-02097-4

**Published:** 2024-01-30

**Authors:** Santosh Kumar Sharma, Devaki Nambiar

**Affiliations:** 1 Statistical Support Officer (Postdoctoral Researcher), University of Limerick, Limerick, Ireland; 2 Healthier Societies, The George Institute for Global Health, New Delhi, India; 3 Faculty of Medicine, University of New South Wales, Sydney, Australia; 4 Prasanna School of Public Health, Manipal Academy of Higher Education, Manipal, India

**Keywords:** Institutional delivery, Benefit incidence, Inequality, South India

## Abstract

**Background:**

Despite a commendable rise in the number of women seeking delivery care at public health institutions in South India, it is unclear if the benefit accrues to wealthier or poorer socio-economic groups. The study’s aim was to investigate at how the public subsidy is distributed among Indian women who give birth in public hospitals in the southern regions.

**Methods:**

Data from the Indian Demographic Health Survey’s fifth wave (NFHS-5, 2019–21) was used in this study. A total of 22, 403 were institutional deliveries across all the southern states of India were included. Out-of-pocket expenditure (OOPE) on childbirth in health institutions was the outcome variable. We used summary statistics, Benefits Incidence Analysis (BIA), concentration index (CI), and concentration curve (CC) were used.

**Results:**

Most women in the lowest, poorest, and medium quintiles of wealth opted to give birth in public facilities. In contrast, about 69% of mothers belonging to highest quintile gave birth in private health institutions. The magnitude of CI and CC of institutional delivery indicates that public sector usage was concentrated among poorer quintiles [CIX: − 0.178; SE: 0.005; *p* < 0.001] and private sector usage was concentrated among wealthier quintiles [CIX: 0.239; SE: 0.006; *p* < 0.001]. Benefit incidence analyses suggest that middle quintile of women received the maximum public subsidy in primary health centres (33.23%), followed by richer quintile (25.62%), and poorer wealth quintiles (24.84%). These pattern in the secondary health centres was similar.

**Conclusion:**

Poorer groups utilize the public sector for institutional delivery in greater proportions than the private sector. Middle quintiles seem to benefit the most from public subsidy in terms of the median cost of service and non-payment. Greater efforts must be made to understand how and why these groups are being left behind and what policy measures can enhance their inclusion and financial risk protection.

**Supplementary Information:**

The online version contains supplementary material available at 10.1186/s12939-024-02097-4.

## Background

The National Health Accounts (NHA) of India, in their most recent report, reveal that the government’s share of the total Gross Domestic Product (GDP) spent on health has increased to 2.16% in 2018-19 from 1.15% in 2013-14 [1–3]. India is still spending the lower proportion of GDP on health, despite a rising trend of public health investment, than the south-east Asian countries, like Thailand and Indonesia, whose proportion of GDP is spent on public health is 3.8% and 2.9%, respectively in 2019 [4]. This relatively low spend is directly related to large and growing levels of catastrophic health expenditure (CHE) and financial hardship in India [5].

In part as a response to these mounting financial burdens, the National (Rural) Health Mission (NRHM, subsequently renamed NHM), was launched by Indian Government, among the most significant reforms setting the country [5–8]. NHM aims to improve primary health– with an initial focus on maternal, neonatal, and child health (particularly institutional deliveries)- through community outreach, fit-for-purpose health personnel, and improving infrastructure for healthcare, particularly in poorer and underserved regions of the country [6, 9, 10]. Since 2005, state and central governments have implemented a number of programs under NHM to widen its remit and make further improvements to health system structure and service design. The aim was to ensure that each person can avail healthcare services that are accessible, equitable, and of quality [6, 11].. A few years after this, in 2008, with an aim to prevent excessive out-of-pocket expenses, and increasing the use healthcare services, the government introduced a national health insurance program called “Rashtriya Swasthya Bima Yojana (RSBY)”.

Several studies suggest mixed impacts of these reforms on utilization of healthcare services and health consequences [5, 12–14]. Healthcare subsidies from the government have led to higher usage and reductions in access disparities [15, 16], although there are important inequality variations by standard of treatment (primary health centres, and hospitals) and kind of services (inpatient, and outpatient) [11, 14]. In addition to a number of other issues including poor enrolment and use, RSBY does not appear to have been successful in lowering OOPE [3].

Notwithstanding these major reforms, as of 2018-19, with 40.6% of the country’s total health spending, India continues to exhibit one of the most elevated rate of OOPE worldwide [1].. The prevalence of CHE for households using private hospitals was 43.99% (defined in this instance as spending 10% or more of total income on health) [3, 17, 18].

In southern Indian states, epidemiological transitions and higher levels of development have been linked to higher expenditure from OOP, CHE, per capita expenditure on public health, and distress funding compared to other regions and states in the union [18, 19]. For instance, in 2018, the prevalence of CHE (defined as health spending above the 10% threshold level,) was highest in Kerala (33.8%) in the country, followed by Andhra Pradesh (23.1%), Karnataka (12.9%), Telangana (11.2%), and Tamil Nadu (20.4%) [18]. The south Indian states had the highest OOP spending on institutional births, with varying distribution. According to NFHS-5 data, it was observed that Kerala (Rs. 26,134) had the highest spending compared to Tamil Nadu (RS. 14,821), Telangana (Rs. 13,758), Andhra Pradesh (Rs. 12,942), and Karnataka (Rs. 11,938) [20].

To address the drawbacks of RSBY, in 2018, the 2017 National Health Policy launched Ayushman Bharat– a broad-based reform platform comprising two components: the Prime Minister’s people’s insurance scheme or the Pradhan Mantri Jan Arogya Yojana (PMJAY) and facility-based comprehensive primary health service enhancement through Health and Wellness Centres (HWC). The financial coverage provided by PMJAY is approximately 17 times more extensive compared to RSBY [21, 22], while HWCs aim to strengthen preventive and essential curative facilities, with an initial focus on seven service packages comprising immunization, maternal and child health, and communicable diseases, and additional service packages related to Non-Communicable Disease (NCD) prevention and management, mental health, injuries and more [23]. The impact of these reforms across population subgroups in India is now an emerging area of interest– and concern.

This is particularly since national averages describing the use of services for birth deliveries in community health facilities conceal huge variability between states and between socioeconomic strata, with the southern states of India (namely, Andhra Pradesh, Karnataka, Kerala, Telangana, Tamil Nadu, Lakshadweep, Puducherry, Andaman & Nicobar Islands) having the dubious distinction of large inequalities. Although the usage of services for maternal care at public health institutions has increased in South India, it is undetermined if potential advantages favour the poor or the wealthy more than others, because little is known about who is benefitting.

Several studies in developing countries, including India, have employed a variety of methods to explore and understand the distributional impacts of public subsidies, including Benefit Incidence Analysis (BIA), concentration curves, and concentration indices [5, 7, 9, 11, 24–26]. Of these, BIA is a method to determine if the subsidies are benefiting the less affluent people in society or the more affluent people. Additionally, it entails determining the financial standing of the facilities, and their distribution in communities [9, 27]. This estimation helps to understand how well governments distribute their finite resources to address the necessities of the underprivileged [9, 27, 28]. Of late, BIA is becoming more prevalent in health economics research [5–7, 9, 11, 15, 24, 29, 30]. At the sub-national level in India, BIA is continuously being used to address particular equality challenges, including the usage of public health or childcare delivery services [6, 10, 11, 31, 32].

With this background, we employed BIA, supplemented with concentration indices, to investigate disparities in how public benefits were distributed among women who gave birth in public health institutions in the southern states of India.

## Data and methods

### Data

Data from the Indian Demographic Health Survey’s fifth wave (NFHS-5, 2019–21) were used. The National Family Health Survey (NFHS) is designed to furnish comprehensive information on the socio-demographic and economic conditions of households, child health, maternal health, sexual and reproductive health, family planning, non-communicable diseases and health care utilization, contraceptive use, disease screening for Indian states and union territories [20]. The multistage stratified sampling design was adopted in NFHS-5. Detailed information on the sampling design and instruments is available elsewhere [20]. The survey collected the information from 724,115 ever-married women aged 15–49, 101, 89 men aged 15–54 from 636,699 households in India. In NFHS-5, the data on OOPE on delivery care was composed through a series of questions by a hospital stay, tests, medicines, transportations, and other costs for the last birth and compensation through the Janani Suraksha Yojana (JSY).

We utilized the data subset which provided specific information on childbirths in the five years before the survey. There were 232, 920 births in total, among them 176,843 were last births, and 155,624 were delivered in medical facilities (i.e., they constitute institutional deliveries). Since this study focused on all South Indian states such as Andhra Pradesh, Andaman & Nicobar Islands, Karnataka, Kerala, Lakshadweep, Puducherry, Telangana, and Tamil Nadu, we restricted our sample to 22,403 mothers in these states who had reported institutional deliveries.

### Variables of the study

We have used a number of variables such as institutional delivery, delivery care at the public health sector (subcentre [SC], Primary Health Centre [PHC]/additional PHC, Urban family Welfare Centre [UFWC]/ Urban Health Post [UHP]/Urban Health Centre [UHC], other public sector health facility, government/municipal hospital, government dispensary, community health centre [CHC]/rural hospital/block PHC), the total cost of delivery, place of residence (rural/urban), educational attainment (less than ten years schooling, 10 & above years of schooling), social group (Scheduled Caste, Scheduled Tribe, Other backward class, Others)^1^, wealth quintile (poorest, poorer, middle, richer, and richest), ANC visits (< 4 visits, ≥ 4 visits), and household size (less than 5, 5 and above)were used in the analyses, based on indicator definitions used in previous studies [3, 5, 17, 18, 26]. The term “institutional birth” refers to the delivery of a child that takes place within a healthcare facility, whether public or private health facility. Primary care was defined as care obtained by subcentre, PHC, UHC/UHP/UFWC, and other public sector health facilities, whereas secondary care was defined as care received from government/municipal hospital, government dispensary, CHC/rural hospital/block PHC. OOPE was used as the outcome variable, defined as delivery care expenditure in a health facilities without reimbursement. In NFHS-5, the mother was asked the following question regarding OOPE to estimate spending during the course of the last birth in the five years prior to the survey: “How much in total did it cost you out of your pocket for this delivery?”

We used a state-specific asset-based wealth measure as a substitution for economic position of households for the southern states of India. Land ownership, drinking water, household durables, sanitary facilities, electricity, type of house, per person number of rooms etc., were used to create the state-specific wealth index. The construction of the state-specific index involved the categorization of the data into binary variables sets and the allocation of indicator weights using principal component analysis (PCA). Five quintiles were created from the resulting state-specific wealth index: poorest, poorer, middle, richer, and richest [35].

### Statistical analysis

The study used descriptive and bivariate analysis, as well as computation of Benefit Incidence Analysis (BIA) scores, Concentration Indices (CIX), and Concentration Curves (CC). Categorical variables, such as residence, maternal education, social group, and household size, were represented in numbers and percentages along with 95% confidence intervals. All proportions were calculated after excluding any missing data.

#### Benefit incidence analysis

To assess the extent of inequality in the distribution of public subsidies for institutional delivery among various socio-economic groups and different types of health centres (public or private), benefit incidence analysis (BIA) was employed. The fundamental principle of BIA is that people from lower socioeconomic strata should benefit from public spending and services provided by the government. As the emphasis on pro-poor health financing grows, benefit incidence analysis (BIA) has become a reliable method for examining the benefits derived from public health funding. Obtaining the precise cost of service for institutional births poses significant challenges when estimating benefit incidence analysis (BIA).

Several studies have used OOPE at private facilities as a substitute to estimate the cost of treatment services in the absence of accurate cost of service [9, 11, 24, 28, 36]. Since, there is a large heterogeneity in the data and the presence of null values, hence mean and mode values would not be suitable. Consequently, we decided to use the median OOPE at private medical facilities as an approximation for the cost of treatment in a public health facility. The following steps were involved to estimate the benefit incidence for childbirth delivery:

1. To assess the socio-economic status, individuals were ranked by wealth and grouped into quintiles.
2. The utilization rate for institutional delivery in public health centres was estimated based on wealth quintiles.
3. The net subsidy was computed by subtracting the median out-of-pocket expenditure (OOP) in public health facilities from the median OOP in private health facilities. This computation assumes that the median OOP can provide an approximate estimation of the actual cost. See below for details.
4. The individual subsidy was calculated by multiplying the utilization rate for each wealth quintile by the net subsidy.
5. The benefit incidence was calculated by determining the percentage contribution of each quintile to the overall subsidy.

The benefit incidence was estimated for a group ‘j’ utilizing institutional delivery service ‘i’ in a public health centre. Mathematical equation of method is:

$$ {\eta }_{j}=\sum {\alpha }_{ij }\frac{{\rho }_{i}}{{\alpha }_{i}}= \sum {\theta }_{ij} {\rho }_{i}$$

Where ${\eta }_{j}$= Benefits of public subsidy utilized by group *j*

${\alpha }_{ij }$= Utilization of delivery care (*i*) by group *j*

${\alpha }_{i}$ = Utilization of delivery care (*i*) by all group

${ \rho }_{i}$= Government net expenditure on delivery care (*i*)

${\theta }_{ij}$ = group *j* share of utilization of delivery care i

OOPE was estimated according to wealth quintile for women delivering at public medical facilities. The information about true expense of childbirth services at the public medical institution was not collected by NFHS-5. Therefore, in accordance with the earlier research, we used OOPE for childbirth services at private medical institutions as the substitutions for the true cost at public medical institutions [6, 9].

To understand the socio-economic inequality in health outcomes, concentration curve (CC) and concentration index (CIX) was used increasingly in public health research [6, 7, 11, 37]. We used Stata’s conindex package to estimate the concentration index. This study examines outcome variables that possess binary characteristics, either ordinal or bounded in nature, rendering them incompatible with rank-dependent measures like the concentration index. Such measures, which gauge relative inequality, do not permit comparisons of differences between individuals [38, 39]. For binary health outcome variables, in large samples, the concentration index will fall between µ-1 and 1-µ, rather than within the usual normal bands. This suggests the need for some form of normalization. Therefore, we used concentration index (CIX) with Erreygers’ correction, a quasi-absolute measure appropriate for binary health outcome [40, 41], which can be written as

$$ Ec=\left(4\mu \right)/\left(b-a\right).C$$

Where, C = standard concentration index, $\mu$ is the mean of the health outcome variable with its range defined $\left(b-a\right)$ (b is the upper bound, and a is the lower bound). A negative value of CIX suggests concentration of health outcome variable in lower levels of socioeconomic status, and a positive value indicates concentration among those who are more affluent.

The CC in our study reflects the cumulative proportions of women according to wealth index against the cumulative proportions of women opted medical facilities for birth delivery. The curve below the line of equality indicates that the women belonging to affluent class of households used health facilities at a higher rate than the lower economic classes of households. Likewise, the concentration curve situated above the equality line indicated that women from economically disadvantaged households had a higher rate of utilizing health facilities for childbirth. Value of CIX varied from − 1 to + 1. There is no inequality, if the value of CIX is zero [42]. Stata®17 was used for all the statistical analysis, using suitable survey weights.

## Results

Table 1 provide the distribution of women according to their background characteristics. About 42.0% (95% CI: 41.8, 42.3) individuals living in urban areas, while 58.0% (95% CI: 57.7, 58.2) in rural areas. About 31.4% (95% CI: 30.7, 32.1) women reported less than ten years of schooling, whereas more than two-thirds (68.6%, 95% CI: 67.9, 69.3) of them had more than ten years of schooling. Regarding social group, 21.7% (95% CI: 21.1, 22.4) individuals identified as Scheduled Caste, 7.4% (95% CI: 7.0, 7.8) as Scheduled Tribe, 58.3% (95% CI: 57.5, 59.0) as Other Backward Classes, and 12.7% (95% CI: 12.2, 13.2) as other social groups. About 58.4% (95% CI: 57.6, 59.2) women adopted public health institutions for birth delivery whereas, 41.6% women adopted private health institutions (95% CI: 40.8, 42.4). Among women who adopted public health institutions, among them, about 46.4% (95% CI, 45.6, 47.2) choose government/municipal hospitals/rural hospitals whereas, about 11.9% (95% CI: 11.5, 12.5) choose subcentres/PHC/UHC/others facilities. It was observed that about 77% women had four or more ANC visit, whereas 21.4% women had less than 4 ANC visits.

**Table 1:** Sample profile of the study population based on NFHS-5, 2019-21 in southern states of India

Variables	N (22,403)	Percentage (%)	95% CI
**Place of residence**			
Urban	9,416	42.0	[41.8, 42.3]
Rural	12,987	58.0	[57.7, 58.2]
**Education level (years)**			
Less than 10	7,032	31.4	[30.7, 32.1]
10 and above	15,371	68.6	[67.9, 69.3]
**Social group**			
Scheduled Caste (SC)	4,862	21.7	[21.1, 22.4]
Scheduled Tribe (ST)	1,651	7.4	[7.0, 7.8]
Other Backward Class (OBC)	13,051	58.3	[57.5, 59.0]
Others	2,839	12.7	[12.2, 13.2]
**Household size**			
Less than 5	8,279	37.0	[36.2, 37.7]
5 and above	14,124	63.1	[62.3, 63.8]
**Place of delivery**			
Public health facility	13,080	58.4	[57.6, 59.2]
Private health facility	9,323	41.6	[40.8, 42.4]
**Level of care at health centres**			
Sub-centres/PHC/UHC/Others^a^	2,683	11.9	[11.5, 12.5]
Government/Municipal/Rural Hospital	10,398	46.4	[45.6, 47.2]
Private health facility	9,323	41.6	[41.9, 42.4]
**Number of ANC visits**			
Less than 4	4,800	21.4	[21.2, 22.5]
4 and more	17,190	76.7	[77.5, 78.8]

^a^Others include additional Primary Healthcare Centre (PHC), Urban Health Post (UHP), Urban Family Welfare Centre (UFWC), Public sector health facility

**Fig. 1:**
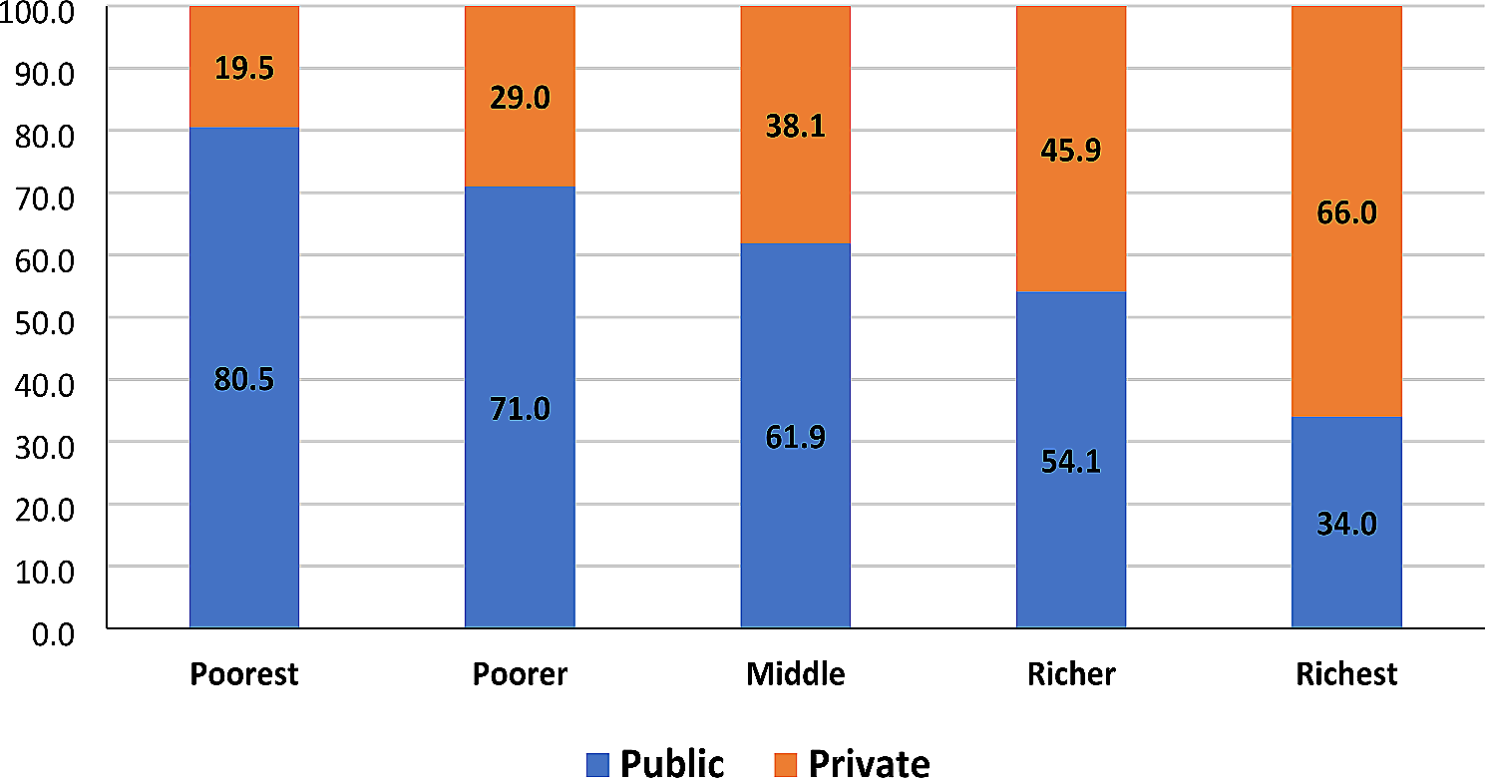
Percentage distribution of institutional delivery by wealth quintile and type of health centre in South India, 2019-21 (*N* = 22,403)

Figure 1 displays the distribution of the institutional delivery according to health care facilities and wealth quintiles in South India. It was observed that as the economic well-being of the households increased, the childbirth in public health institutions decreased: a negative gradient. In contrast, a positive economic gradient was seen for childbirth in private health institutions. For instance, about 80.5% births were delivered in public health institutions and 19.5% in private facilities of all the childbirths in lowest wealth quintile. On the other hand, among women in highest wealth quintile, over two-thirds opted for private health institutions for childbirth. Overall, most women belonging to the bottom three quintiles utilized childbirth facilities in public medical institutions.

**Fig. 2:**
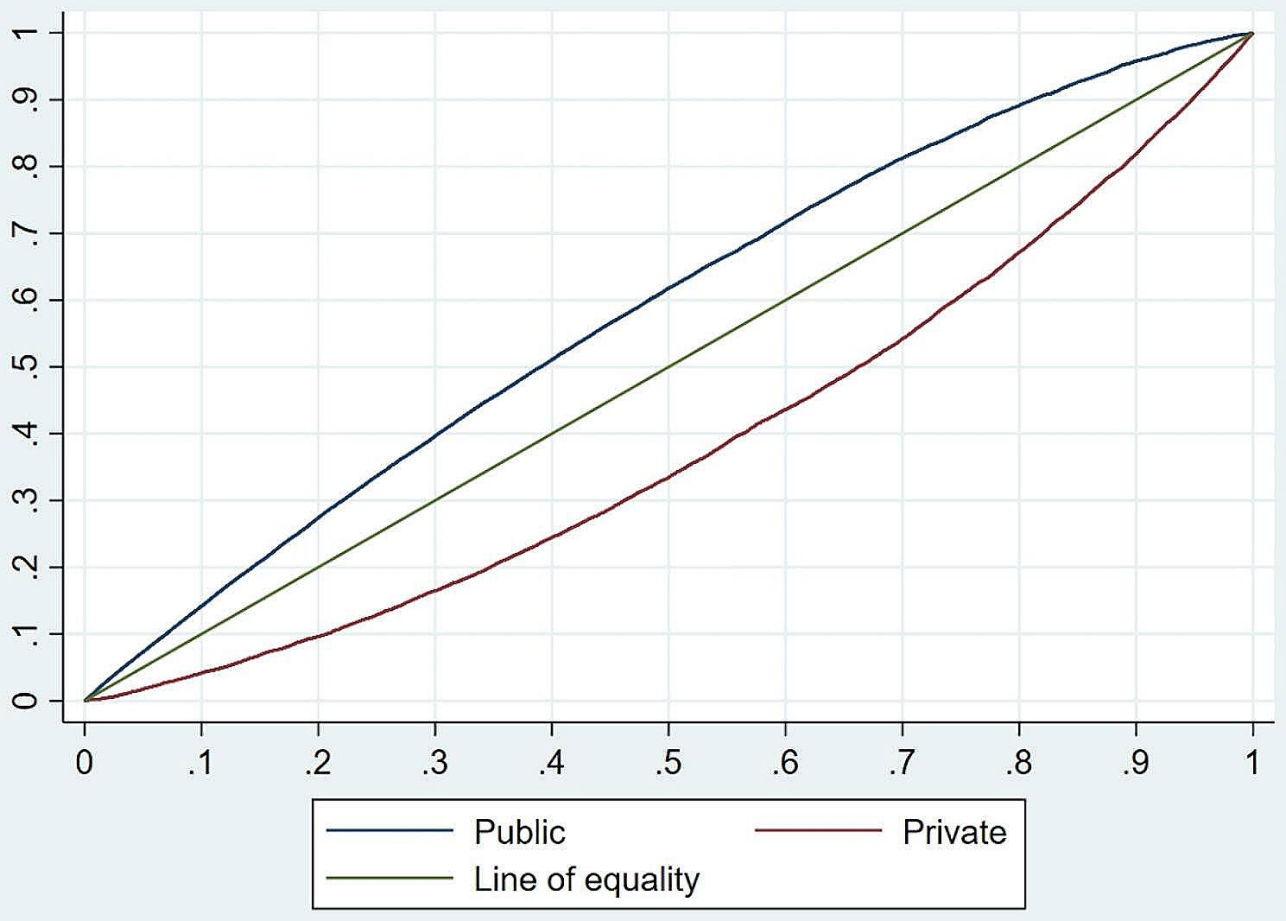
Concentration curve for mothers using delivery services at public and private health facility in South India, 2019-21

Figure 2 display the CC for women who delivered childbirth in public and private health institutions. The CC positioned above the equality line, indicating a pro-poor distribution among women who used public health institutions for childbirth, In contrast, women who utilized private health institutions for childbirth exhibited a CC situated below the equality line, suggesting a pro-rich concentration. We also computed concentration indices by other dimensions of inequality such as place of residence, education, household size, social group, and number of ANC visits (See supplementary Table 1), finding that across the board, public sector usage was concentrated among poorer quintiles and private sector usage was concentrated among wealthier quintiles. This finding was statistically significant.

**Fig. 3:**
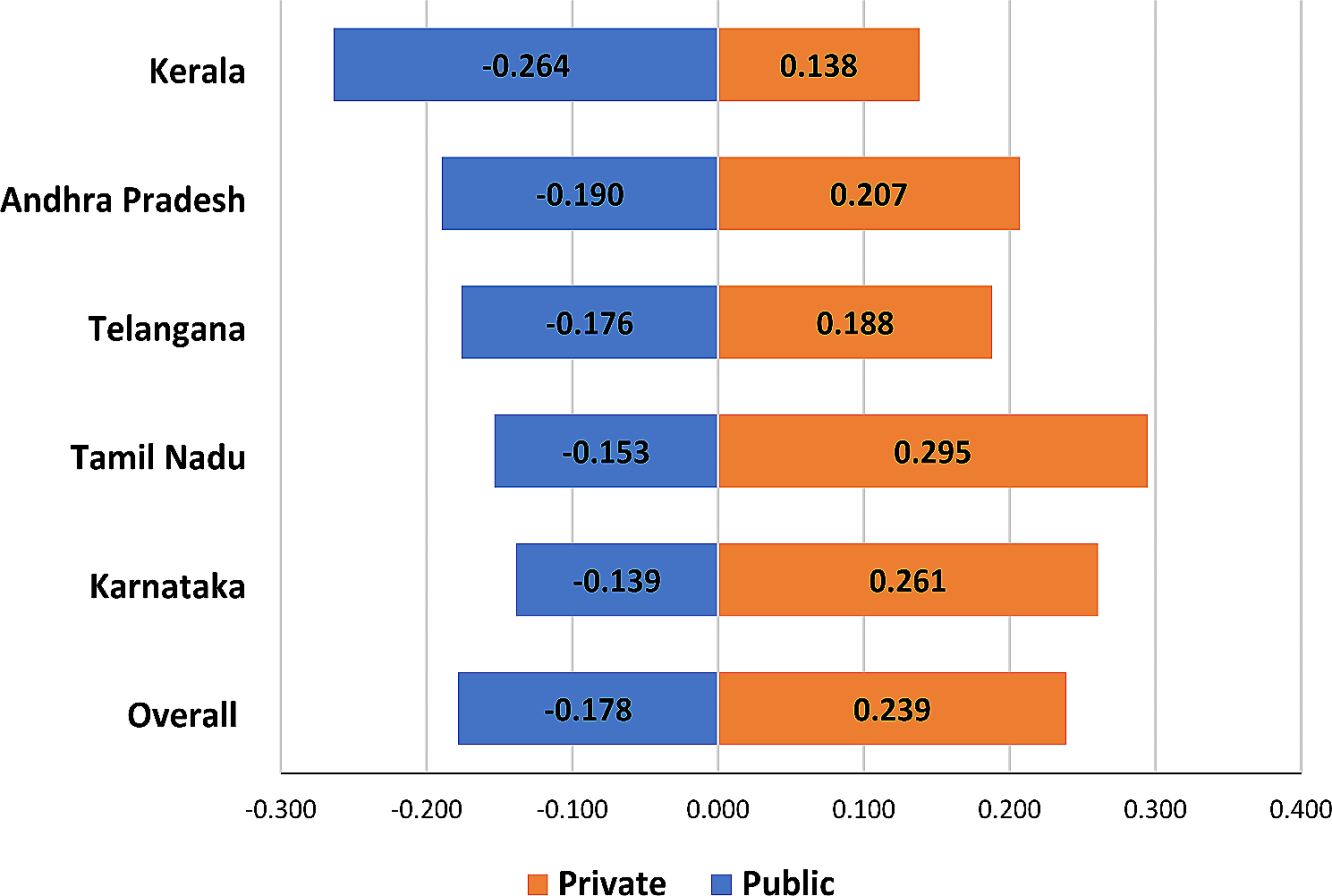
Concentration Index of institutional delivery by public and private facility in selected states of South India, 2019-21

Figure 3 illustrates the CI for women who delivered childbirth at public and private health institutions in South India. Overall, for all southern states, the magnitude of the concentration index (CIX = -0.178; SE: 0.005) indicated a negative value, suggesting that women who gave birth in healthcare facilities were concentrated in public health institutions and was negative for all the southern states. In contrast, for all the southern states, the positive CIX value (CIX = 0.239; SE: 0.006) suggested that women who gave birth in health facilities were concentrated in private health centres. In Kerala, the higher and negative magnitude of CIX (CIX = -0.264; SE:0.019) indicated that poorer women opted for public health institutions for childbirth. Similarly, a higher and positive magnitude of CI indicated that childbirth in private health institutions was concentrated among women of wealthier households in Tamil Nadu (CIX = 0.295; SE:0.017), Karnataka (CIX = 0.261; SE:0.016), and Andhra Pradesh (CIX = 0.207; SE:0.015). Overall, public sector use was strongly concentrated among poorer populations in Kerala, and with a somewhat smaller margin, private sector use was concentrated in wealthier population groups (suggesting that use of private sector is also prevalent among poorer groups). In Andhra Pradesh and Telangana where the same overall pattern was seen, margins of concentration in the public and private sectors were similar. Finally, in Karnataka and Tamil Nadu, the CIX values for private sector use were higher, suggesting that use is much greater concentration of private sector use in wealthier population groups.

Table 2 presents wealth quintile-based distribution of benefit incidence, OOPE, and utilization rate pertaining to childbirth in health facilities, along with various types of public medical institutions in South India. Among the different wealth quintiles, The usage rate of primary health institutions for childbirth was highest in poorest quintile (32.69%) and lowest in the richest quintile (7.70%). Similarly, for secondary health institutions, it ranged from 25.79% for poorest quintile to 11.66% for the richest quintile. It was observed that, the usage rate of any public health institutions for childbirth was highest in poorest quintile of women (27.26%), and lowest in richest quintile (10.81%). Further, by employing the total median OOPE for childbirth in private medical institutions as the substitution for the facility cost, the concentration of public subsidy across all public medical facilities was primarily observed in the poorest quintile of wealth status. Public funding for primary health institutions in South India during 2019–21 was greatest for the poorest wealth quintile (32.78%), then higher for the poorer quintile (25.02%), then lower for the richer quintile of wealth status (15.13%), and minimum for the richest wealth quintile (7.45%). Among the wealth quintiles, the highest advantage of public funding for childbirth in secondary health institutions was observed in the poorest quintile (25.79%), followed by the poorer quintile (23.73) and the middle quintile (20.87%), with the lowest benefit occurring in the richest quintile (11.66%). When considering the quintile-specific median cost of care in private health institutions, the pattern of the advantage of public subsidy remained consistent across all public health institutions in South India. (See supplementary Table S2).

**Table 2:** Utilization rate, out-of-pocket payment (OOP in INR), and benefit incidence on institutional delivery by wealth quintile and level of care in South India, 2019-21

Type of Public health centre	Wealth quintile	Number people utilizing public health service (1)	Utilization rate (2)	Median OOP in public health service (3)	Median cost of service in private health centre (4)	Net subsidy at public health centre (5 = 4 − 3)	Individual subsidy (6 = 5*2)	Benefit incidence (%) (7)	N	
Primary: Sub-centres/PHC/ UHC/Others^a^	Poorest	925	0.3269	2000	30,000	28,000	9152	32.78	3622	
	Poorer	706	0.2495	2000	30,000	28,000	6985	25.02	3,188	
	Middle	554	0.1958	2000	30,000	28,000	5481	19.63	2,737	
	Richer	427	0.1509	2000	30,000	28,000	4225	15.13	2,304	
	Richest	218	0.0770	3000	30,000	27,000	2080	7.45	1,437	
	Total	2,830					27,923		13,288	
Secondary: Government/Municipal /Rural Hospital	Poorest	2697	0.2579	3000	30,000	27,000	6963	25.79	3622	
	Poorer	2,482	0.2373	3000	30,000	27,000	6408	23.73	3,188	
	Middle	2,183	0.2087	3000	30,000	27,000	5636	20.87	2,737	
	Richer	1,877	0.1795	3000	30,000	27,000	4846	17.95	2,304	
	Richest	1,219	0.1166	3000	30,000	27,000	3147	11.66	1,437	
	Total	10,458					27,000		13,288	
Any public health facility	Poorest	3622	0.2726	2600	30,000	27,400	7469	27.55	3857	
	Poorer	3,188	0.2399	3000	30,000	27,000	6478	23.90	4141	
	Middle	2,737	0.2060	3000	30,000	27,000	5561	20.51	4275	
	Richer	2,304	0.1734	3000	30,000	27,000	4682	17.27	4678	
	Richest	1,437	0.1081	3000	30,000	27,000	2920	10.77	5453	
	Total	13,288					27,109		22,403	

Table 3 shows the wealth quintile-based distribution of benefit incidence, OOPE, and utilization rate pertaining to childbirth in health facilities based on place of residence, education and social caste groups in South India. It was observed that the allocation of public subsidies varied across poorer to middle-wealth quintile groups for each selected socio-demographic and socioeconomic characteristic. Urban-dwelling women in the richer quintile received the highest share of benefits (28.23%), followed by the richest (25.43%) and middle quintile (20.15%). Benefit accrued the least to women belonging to lowest wealth quintile (10.35%). In the case of rural areas of South India, women from the poorest quintile received the largest percentage of benefits (34.47%), then the women from the poorer quintile (27.10%), while women from wealthier groups received the lowest share (4.72%). The usage rate of public health institutions was largest in poorest wealth quintile of women (44.33%) who had education below 10 years, and lowest in the richest quintile of women (3.56%). Poorest wealth quintile of women who had education below 10 years (44.61%) received highest benefits of public subsidy, then the poorer quintile of women (25.70%), whereas, richest quintile of women (3.58%) received the lowest benefits. Similarly, middle quintile of women who had 10 and above years of education (23.25%), received the highest benefits of public subsidy, then the poorer (22.77%) and richer quintile of women (22.51%), whereas poorest (15.76%) and richest quintile of women (15.71%) benefited the lowest.

**Table 3:** Utilization rate, out-of-pocket payment (OOP in INR), and benefit incidence by place of residence, educational attainment and social group on institutional delivery in South India, 2019-21

Variables	Wealth quintile	Number people utilizing public health service (1)	Utilization rate (2)	Median OOP in public health service (3)	Median cost of service in private health centre (4)	Net subsidy at public health centre (5 = 4 − 3)	Individual subsidy (6 = 5*2)	Benefit incidence (%) (7)	N
Urban	Poorest	402	0.1041	3000	30,150	27,150	2826	10.35	3622
	Poorer	609	0.1577	2700	30,150	27,450	4329	15.85	3,188
	Middle	786	0.2035	3100	30,150	27,050	5505	20.15	2,737
	Richer	1077	0.2789	2500	30,150	27,650	7711	28.23	2,304
	Richest	988	0.2558	3000	30,150	27,150	6946	25.43	1,437
	Total	3862					27,316		13,288
Rural	Poorest	3220	0.3416	2530	28,000	25,470	8701	34.47	3622
	Poorer	2579	0.2736	3000	28,000	25,000	6840	27.10	3,188
	Middle	1951	0.2070	2600	28,000	25,400	5257	20.83	2,737
	Richer	1227	0.1302	3000	28,000	25,000	3254	12.89	2,304
	Richest	449	0.0476	3000	28,000	25,000	1191	4.72	1,437
	Total	9426					25,243		13,288
Education less than 10 year	Poorest	2377	0.4433	2500	24,000	21,500	9531	44.61	3622
	Poorer	1382	0.2577	2700	24,000	21,300	5490	25.70	3,188
	Middle	893	0.1665	3000	24,000	21,000	3497	16.37	2,737
	Richer	519	0.0968	2500	24,000	21,500	2081	9.74	2,304
	Richest	191	0.0356	2500	24,000	21,500	766	3.58	1,437
	Total	5362					21,365		13,288
Education 10 or more year	Poorest	1245	0.1571	2900	30,000	27,100	4257	15.76	3622
	Poorer	1806	0.2279	3000	30,000	27,000	6152	22.77	3,188
	Middle	1844	0.2327	3000	30,000	27,000	6282	23.25	2,737
	Richer	1785	0.2252	3000	30,000	27,000	6081	22.51	2,304
	Richest	1246	0.1572	3000	30,000	27,000	4245	15.71	1,437
	Total	7926					27,016		13,288
Scheduled Caste	Poorest	1258	0.3486	2700	25,400	22,700	7913	35.16	3622
	Poorer	1032	0.2860	3000	25,400	22,400	6405	28.46	3,188
	Middle	678	0.1879	3000	25,400	22,400	4208	18.70	2,737
	Richer	448	0.1241	3000	25,400	22,400	2781	12.36	2,304
	Richest	193	0.0535	3000	25,400	22,400	1198	5.32	1,437
	Total	3609					22,505		13,288
Scheduled Tribe	Poorest	592	0.4415	2200	23,500	21,300	9403	44.25	3622
	Poorer	301	0.2245	3000	23,500	20,500	4601	21.65	3,188
	Middle	217	0.1618	2000	23,500	21,500	3479	16.37	2,737
	Richer	146	0.1089	2000	23,500	21,500	2341	11.02	2,304
	Richest	85	0.0634	1000	23,500	22,500	1426	6.71	1,437
	Total	1341					21,251		13,288
Other Backward Class (OBC)	Poorest	1527	0.2134	2500	30,000	27,500	5867	21.59	3622
	Poorer	1624	0.2269	2700	30,000	27,300	6195	22.80	3,188
	Middle	1570	0.2194	3000	30,000	27,000	5923	21.80	2,737
	Richer	1462	0.2043	3000	30,000	27,000	5515	20.30	2,304
	Richest	974	0.1361	3000	30,000	27,000	3674	13.52	1,437
	Total	7157					27,175		13,288
Other castes	Poorest	245	0.2075	3700	35,000	31,300	6493	20.75	3622
	Poorer	231	0.1956	3400	35,000	31,600	6181	19.75	3,188
	Middle	272	0.2303	4500	35,000	30,500	7025	22.44	2,737
	Richer	248	0.2100	4000	35,000	31,000	6510	20.80	2,304
	Richest	185	0.1566	2500	35,000	32,500	5091	16.27	1,437
	Total	1181					31,299		13,288

The pattern of public subsidy benefits across social groups exhibited a similar trend. For instance, the usage rate of public health institutions for childbirth was higher among SC women belonging to poorest quintile (34.86%), and poorer quintile (28.60%) than the richest quintile (5.35%). It was also observed that SC women in poorest quintile (35.16%), and poorer quintile (28.46%), received the highest benefits of public subsidy, whereas, it was lowest among SC women in the richest quintile (5.32%). Similarly, among mothers belonging to ST, poorest quintile of women (44.25%) received highest benefits from public subsidy, then the poorer quintile (21.65%), whereas richest quintile of women (6.71%) received the lowest benefits.

We also looked at actual out-of-pocket expenditures to determine how this concorded with benefit incidence (Table 4). In South India, approximately 8.2% of the respondents received delivery care without any payment, with the percentage varying from 10.5% in the poorest quintile of wealth status to 4.5% in the richest quintile of wealth of status. Out of the women who utilized services at subcentres/PHC/UHC/Others, 16.0% did not incur any costs for delivery care. Specifically, 22.7% of those belongs the middle quintile of wealth status did not pay, while 10.5% of women from the affluent class did not pay. Likewise, among women who accessed services from Government/Municipal/Rural Hospital health centres, 11.9% did not incur any costs for delivery care. This percentage varied from 12.6% in the poorer wealth quintile to 10.7% in the richest wealth quintile. Where any public health facility was used, 12.7% of women did not incur any cost for delivery care, varying from 14.0% in the middle wealth quintile to 10.6% in the richest wealth quintile.

**Table 4:** Percent distribution of mothers who paid and did not pay for institutional delivery by wealth quintile and type of health centres in South India, 2019-21

Wealth quintile	a. Sub-centres/PHC /UHC/Others^a^			b. Government/Municipal /Rural Hospital			c. Any public health facility (a or b)			d. Private health facility			Overall				
	Paid (%)	Didn’t pay (%)	N	Paid (%)	Didn’t pay (%)	N	Paid (%)	Didn’t pay (%)	N	Paid (%)	Didn’t pay (%)	N	Paid (%)		Didn’t pay (%)	N	
Poorest	84.9	15.1	925	89.0	11.0	2697	87.9	12.1	3622	96.3	3.7	859	89.5		10.5	3857	
Poorer	84.6	15.4	706	87.4	12.6	2,482	86.8	13.2	3,188	97.3	2.7	1293	89.9		10.1	4141	
Middle	77.3	22.7	554	87.6	12.4	2,183	85.6	14.0	2,737	98.4	1.6	1,743	90.5		9.5	4275	
Richer	88.6	14.5	427	87.7	12.3	1,877	87.3	12.7	2,304	98.3	1.7	2,177	92.3		7.7	4678	
Richest	89.5	10.5	218	89.3	10.7	1,219	89.4	10.6	1,437	98.7	1.3	3,043	95.5		4.5	5453	
**Total**	**84.0**	**16.0**	**2,830**	**88.2**	**11.9**	**10,458**	**87.3**	**12.7**	**13,288**	**98.2**	**1.8**	**9,115**	**91.8**		**8.2**	**22,403**	

^a^Others include additional Primary Healthcare Centre (PHC), Urban Health Post (UHP), Urban Family Welfare Centre (UFWC), Public sector health facility

## Discussion

India’s NHM has made some gains in improving healthcare access, utilization, and population-level maternal and child health outcomes through a major rehaul of service delivery, human resources, and other building blocks [5, 9, 19, 25].. We sought to assess how equitable progress on this path has been using the proxy of institutional delivery, assessing the benefit incidence across quintiles, and comparing the public and private sectors using the most recent and comprehensive large-scale population-based fifth round of Indian Demographic Health Survey (NFHS-5, 2019-21).

The key results of this study can be summarized as follows:

First, most women belonging to poorest, poorer, and middle quintiles opted public health institutions for childbirth in southern states of India, whereas a greater proportions of women belonging to the richer and the richest wealth quintile used private health institutions for childbirth. Concentration curves corroborated this: women using public health institutions for childbirth were pegged above the equality line, indicating that public health services for childbirth was concentrated among poor. Conversely, the curve below the equality line, indicating a disproportionate concentration of private health services among the women belonging to affluent class of households.

Second, the variation in concentration index by southern states of India revealed that in Kerala, the concentration of private sector institutional delivery care services is less than in all other states, with Andhra Pradesh and Telangana having matched magnitudes of the public (among the poor) and private sector (among the rich) utilization. In Tamil Nadu, private sector institutional deliveries are strongly pro-rich concentrated.

Third, public subsidy distribution for institutional delivery care services was found to be highest in the poorest wealth quintile in public health facilities of South India. Women from the poorest wealth quintile, living in rural areas, with less than ten years of education, belonging to SC and ST benefitted the most from public subsidy, then poorer quintile of wealth status. Overall, it was concerning that the poorest quintile of women, particularly in urban settings, had the lowest public subsidy benefit incidence in South India, pointing towards critical exclusions.

Finally, we noted in tandem with benefit incidence accruing quite heavily for middle-income quintiles, larger proportions were not paying for institutional delivery in these groups as compared to poorer quintiles, particularly in public facilities. This suggests that the inequitable incidence of the benefit of public subsidy may in part, be offsetting the costs of institutional delivery, but not among those with the greatest economic disadvantage [5, 9, 11, 26].

It was expected that the use of the public sector would be concentrated among poorer sub-populations in these states, and the inverse, that private sector use would be concentrated among wealthier quintiles. This is a global phenomenon, particularly in countries that have a “mixed health systems syndrome” [43].

We found intriguing patterns of variation in institutional delivery across southern Indian states: while the overall pattern was that public sector use was concentrated among the poor and private sector use among the wealthy, Kerala seemed to have the possibility of less concentration of use in poorer populations, while this was not the case in Tamil Nadu or Karnataka. With state-level schemes that provide subsidies for the use of the private sector, including for maternity care [44], it is possible that for deliveries, families in Kerala are opting for the private sector in greater relative proportions than in the other southern states. Interpretation of this finding is somewhat tricky, however– is this finding encouraging or troubling? On the one hand, we do not want poorer populations to have restrictions in access to care– be it in the public or private sector. At the same time, we do not wish poorer populations to incur additional financial stress, which is much more likely in the private sector. The inverse is also worth considering, i.e., since the magnitude of concentration in the private sector, it is possible that greater proportions of wealthier groups are using the public sector for deliveries. This, to some extent, feels paradoxical and warrants further study.

We also found greater benefit incidence among middle quintiles. Gita and Iyer (2012) suggested in their paper that the importance of moving beyond the conventional emphasis on well-known and often pronounced disparities among groups situated at the opposite ends of the multi-dimensional socioeconomic spectrum. In contrast to the groups situated at the extremes, those in the middle exhibit a combination of both advantages and disadvantages. The strategies they employ to mitigate their disadvantages while capitalizing on their advantages can be intricate and diverse [45]. Our findings suggest that public subsidy benefits may accrue to these quintiles and could be related to greater non-payment for institutional deliveries. This warrants additional correlational analysis and deeper qualitative inquiry.

It is troubling that even in southern Indian states, the poorest of the poor do not seem to be getting the benefit of existing schemes. The lack of benefit incidence may be in part due to foregone care/non-use of facilities for delivery [46, 47]. Many studies show that institutional deliveries are low among the poorest wealth quintiles [5, 47–51]. This is a critical area of focus in policymaking in particular.

### Limitations

The study provides empirical evidence on the level of inequality in the allocation of public subsidies for institutional delivery using BIA in South India. Nevertheless, there are some limitations that require attention. First, there could have been some recall bias, given that we employed self-reported information from the NFHS to gauge usage trends, OOP expenditure, and benefit incidence. In addition, the survey didn’t cover the indirect costs related to institutional delivery. Second, the median cost of services in private health care facilities served as our proxy for the price of services in public health facilities. A thorough costing assessment might make the argument for the actual scenario more convincing.

## Conclusion

Our study using NFHS-5 data for five southern Indian states found that poorer groups utilize the public sector in greater concentration for institutional delivery than the private sector. However, the urban poor seem to be left behind in the benefit incidence that should accrue as a result. Middle quintiles seem to benefit the most from public subsidy in terms of the median cost of service and non-payment. Further research may shed light on how and why these groups are being left behind and what policy measures could enhance their inclusion and financial risk protection.

### Electronic supplementary material

Below is the link to the electronic supplementary material.

Supplementary Material 1

## Data Availability

The public repository of the Demographic and Health Survey contains all the data utilized in the study (DHS). The following URL, which needs registration, can be used to access the data: https://dhsprogram.com/data/datasetadmin/index.cfm.

## Footnotes

1 According to article numbers 341 and 342 of the Constitution of India, the President of India acknowledge the tribal and caste groups as the Scheduled Tribe’ and ‘Scheduled Caste’ respectively [33]. the Government of India use the term ‘Backward Class’ to categorize groups that face educational or social disadvantages [34].

## Abbreviations

NHA: National Health Accounts
GDP: Gross Domestic Product
CHE: Catastrophic Health Expenditure
UHC: Universal Health Coverage
OOPE: Out-of-Pocket Expenditure
RSBY: Rashtriya Swasthya Bima Yojana
PMJAY: Pradhan Mantri Jan Arogya Yojana
HWC: Health and Wellness Centres
BIA: Benefit Incidence Analysis
NFHS: National Family Health Survey
JSY: Janani Suraksha Yojana
CI: Concentration Indices
CC: Concentration Curves
PHC: Primary Health Centre
PSU: Primary Sampling Unit
NHM: National Health Mission
SES: Socioeconomic status
