# Supplementary material for: Are institutional deliveries equitable in the southern states of India? A benefit incidence analysis
Source: Int J Equity Health. 2024 Jan 30;23:17. doi: 10.1186/s12939-024-02097-4 (PMC10829246; doi:10.1186/s12939-024-02097-4)
Supplement: Supplementary file 1 — Supplementary Material 1 [file 12939_2024_2097_MOESM1_ESM.docx]

**Supplementary file**

| **Table S1: Concentration index for institutional delivery by selected covariates in South India, 2019-21** | | | | | | |
| --- | --- | --- | --- | --- | --- | --- |
|  | **Public health facility** | | | **Private health facility** | | |
|  | **CIX** | **Std. error** | **p-value** | **CIX** | **Std. error** | **p-value** |
| **Place of residence** |  |  |  |  |  |  |
| Urban | -0.203 | 0.009 | 0.000 | 0.208 | 0.009 | 0.000 |
| Rural | -0.124 | 0.005 | 0.000 | 0.220 | 0.009 | 0.000 |
| **Education** |  |  |  |  |  |  |
| Less than 10 years | -0.061 | 0.005 | 0.000 | 0.172 | 0.015 | 0.000 |
| 10 and above years | -0.182 | 0.006 | 0.000 | 0.193 | 0.007 | 0.000 |
| **Household size** |  |  |  |  |  |  |
| Less than 5 | -0.157 | 0.007 | 0.000 | 0.223 | 0.010 | 0.000 |
| 5 and above | -0.171 | 0.005 | 0.000 | 0.238 | 0.007 | 0.000 |
| **Social group** |  |  |  |  |  |  |
| SC | -0.073 | 0.008 | 0.000 | 0.207 | 0.022 | 0.000 |
| ST | -0.121 | 0.013 | 0.000 | 0.286 | 0.031 | 0.000 |
| OBC | -0.166 | 0.006 | 0.000 | 0.191 | 0.007 | 0.000 |
| Others | -0.252 | 0.013 | 0.000 | 0.226 | 0.012 | 0.000 |
| **Number of ANC visits** | |  |  |  |  |  |
| Less than 4 | -0.150 | 0.009 | 0.000 | 0.236 | 0.014 | 0.000 |
| 4 and more | -0.168 | 0.005 | 0.000 | 0.231 | 0.007 | 0.000 |
| Overall | -0.166 | 0.005 | 0.000 | 0.232 | 0.006 | 0.000 |

| **Table S2: Utilization rate, out-of-pocket payment (OOP in US$), and benefit incidence on institutional delivery by wealth quintile and level of care in South India, 2019-21** | | | | | | | | | |
| --- | --- | --- | --- | --- | --- | --- | --- | --- | --- |
| **Type of Public health centre** | **Wealth quintile** | **Number people utilizing public health service (1)** | **Utilization rate (2)** | **Median OOP in public health service (3)** | **Median cost of service in private health centre (4)** | **Net subsidy at public health centre (5=4-3)** | **Individual subsidy (6=5*2)** | **Benefit incidence (7)** | **N** |
| Primary: Sub-centres/PHC/  UHC/Others^a^ | Poorest | 925 | 0.3269 | 2000 | 22000 | 20000 | 6537 | 23.41 | 3622 |
|  | Poorer | 706 | 0.2495 | 2000 | 25000 | 23000 | 5738 | 20.55 | 3,188 |
|  | Middle | 554 | 0.1958 | 2000 | 26300 | 24300 | 4757 | 17.04 | 2,737 |
|  | Richer | 427 | 0.1509 | 2000 | 30000 | 28000 | 4225 | 15.13 | 2,304 |
|  | Richest | 218 | 0.0770 | 3000 | 33500 | 30500 | 2349 | 8.41 | 1,437 |
|  | Total | 2,830 |  |  |  |  | 23606 |  | 13,288 |
| Secondary: Government/Municipal  /Rural Hospital | Poorest | 2697 | 0.2579 | 3000 | 22000 | 19000 | 4900 | 18.15 | 3622 |
|  | Poorer | 2,482 | 0.2373 | 3000 | 25000 | 22000 | 5221 | 19.34 | 3,188 |
|  | Middle | 2,183 | 0.2087 | 3000 | 26300 | 23300 | 4864 | 18.01 | 2,737 |
|  | Richer | 1,877 | 0.1795 | 3000 | 30000 | 27000 | 4846 | 17.95 | 2,304 |
|  | Richest | 1,219 | 0.1166 | 3000 | 33500 | 30500 | 3555 | 13.17 | 1,437 |
|  | Total | 10,458 |  |  |  |  | 23386 |  | 13,288 |
| Any public  health facility | Poorest | 3622 | 0.2726 | 2600 | 22000 | 19400 | 5288 | 19.51 | 3857 |
|  | Poorer | 3,188 | 0.2399 | 3000 | 25000 | 22000 | 5278 | 19.47 | 4141 |
|  | Middle | 2,737 | 0.2060 | 3000 | 26300 | 23300 | 4799 | 17.70 | 4275 |
|  | Richer | 2,304 | 0.1734 | 3000 | 30000 | 27000 | 4682 | 17.27 | 4678 |
|  | Richest | 1,437 | 0.1081 | 3000 | 33500 | 30500 | 3298 | 12.17 | 5453 |
|  | Total | 13288 |  |  |  |  | 23345 |  | 22403 |
